# Supplementary material for: The Impact of Training Time on Understanding the Treatment of Adolescent Idiopathic Scoliosis According to SOSORT International Guidelines: An Online Survey Study
Source: Children (Basel). 2022 Oct 31;9(11):1673. doi: 10.3390/children9111673 (PMC9688895; doi:10.3390/children9111673)
Supplement: Supplementary file 1 [file children-09-01673-s001.zip › children-1915174-supplementary.pdf]

Supplementary material

Characteristics of the participants

1- Gender
Female ( )
Male ( )

2- Age (Years) _______

3- University
Public ( )
Private ( )

4- Academic training:
Bachelor ( )
Specialist ( )
Master ( )
Doctor ( )

5- Clinical Experience (Years) _______

6- Region of operation
North ( )
Northeast ( )
Central West ( )
South ( )
South-east ( )

7- Place of work
Private office or clinic ( )
Public Service ( )
Home care ( )
Fitness centre ( )
Hospital ( )
Other ( )

8- Number of patients with idiopathic scoliosis (per semester)
1 to 5 ( )
5 to 10 ( )
10 or more ( )

9- Do you use any specific technique for treatment?
Yes ( )
No ( )
Which? __________________________

Questionnaire

1- What is idiopathic scoliosis?
a) Three-dimensional deformity found predominantly in childhood and puberty.
b) Abnormal lateral curve in the spine.
c) Most common abnormal deformity found in the second dimension.
d) None of the above.
e) I don't know.

2- What are the causes of idiopathic scoliosis?
a) It is caused by a congenital, vertebral or rib malformation.
b) Fetal malformation in the period of 3 to 6 weeks of pregnancy.
c) This is a structural pathology without a definite cause.
d) Idiopathic scoliosis has multifactorial origins.
e) I don't know.

3- When usually idiopathic scoliosis develops?
a) Idiopathic scoliosis develops in adulthood.
b) Malformation of the spine occurs in the uterus, during the period from 3 to 6 weeks.
c) Idiopathic scoliosis develops most often in childhood and adolescence.
d) Idiopathic scoliosis occurs due to a compensatory disorder.
e) I don't know.

4- What is the prevalence of idiopathic scoliosis?
a) Approximately 20% of cases.
b) Approximately 60% of cases.
c) Approximately 80% of cases.
d) Approximately 40% of cases.
e) I don't know.

5- When is the diagnosis confirmed?
a) The Cobb angle is 20° or greater.
b) When the patient has a morphological change in the rib and/or lateral abnormal curvature of the spine.
c) The patient presents asymmetry of the levels of the iliac crest.
d) Cobb angulation is greater than 10° and associated with axial rotation.
e) I don't know.

6- What does the sign of Risser represent in the radiography?
a. Degree of bone maturation
b. Associated with the degree of scoliosis
c. Excludes idiopathic scoliosis
d. None of the above.
e. I don't know.

7- What is the Adams test?
a) This is a test that aims to evaluate the physiological curvature of the patient's spine. Being noticed when it presents an abnormality from the use of the scoliometer.
b) This is a test that evaluates the level of the iliac crests, in order to identify whether they present a gap, which could indicate the presence of a scoliosis.
c) This is an instrument used to identify the Cobb angle more quickly and accurately, reducing the time taken to complete the patient's diagnosis.
d) None of the above.
e) I don't know.

8 - How is the referral for the evaluation of the physiotherapist?
a) Direct referral from the doctor after the patient reports back pain.
b) Clinical examination, family history and results of radiographic examinations with Cobb angle above 10.
c) Patients who present abnormality of the curvature of the spine are directly referred to the physiotherapist for the kinetic-functional diagnosis, which are analyzed visually.
d) None of the above.
e) I don't know.

9- When should conservative treatment be recommended?
a. 10-45 degrees.
b. 25-50 degrees.
c. It is recommended at any stage.
d. None of the above.
e. I don't know.

10- What are the goals of conservative treatment?
a) Align the patient's spine so that he can use the next stage of treatment, the vest.
b) Increase the Cobb angle of the patient by up to 20.
c) Work on aesthetics, quality of life, functional disability, back pain, reduction of the angle of Cobb and the psychological well-being of the patient.
d) None of the above
e) I don't know.

11- When is the use of a brace recommended?
a) It is recommended when the primary curve is between 5' and 10 ^{3} at the Cobb angle.
b) It is recommended for the treatment of functional scoliosis.
c) It is recommended when the primary curve is equal to or greater than 20 ind at the Cobb angle.
d) None of the above.
e) I don't know.

12- What are the recommendations on physiotherapeutic exercises applied to these patients in order to prevent the progression of idiopathic scoliosis?
a) It is recommended that physiotherapeutic exercises direct to three-dimensional self-correction of the spine, stabilization of corrected posture and patient education during activities of daily living.

b) Group treatments are best used to prevent the progression of scoliosis curvature.
c) Treatment should be done alternately, starting from the beginning with a high intensity.
d) Extension exercises are recommended to prevent the progression of the curvature of the spine.
e) I don’t know.

13- What are the recommendations for patients who have scoliosis and practice physical activity?
a) Stop activities, to avoid increased curvature of scoliosis.
b) Give continuity in physical activity, because it allows an improvement in psychological and social well-being.
c) Change physical activity, always prioritizing activities such as swimming and ballet.
d) None of the above.
e) I don’t know.

14- In your opinion, what conservative treatment method would you recommend for the treatment of idiopathic scoliosis?
a. Method Scientific Exercises Approach to Scoliosis (SEAS)
b. Global Postural Re-education (RPG)
c. Spinal manipulation
d. Specific Exercises for Scoliosis
e. None of the above

15- What physical activity would benefit scoliosis patients (in your opinion)?
a. Yoga
b. Swimming
c. Pilates
d. Ballet
e. I don’t know
